# DNA Methyltransferase1 (DNMT1) Isoform3 methylates mitochondrial genome and modulates its biology

**DOI:** 10.1038/s41598-017-01743-y

**Published:** 2017-05-08

**Authors:** Sunil Kumar Saini, Kailash Chandra Mangalhara, Gopinath Prakasam, R. N. K. Bamezai

**Affiliations:** National Centre of Applied Human Genetics, School of Life Sciences, Jawaharlal Nehru University, New Delhi, 110067 India

## Abstract

Here we demonstrate localization of the isoform3 of DNA Methyltransferase1 (DNMT1) enzyme to mitochondria, instead of isoform1 as reported earlier. The fused DNMT1-isoform1, reported earlier to localize in mitochondria, surprisingly showed its exclusive presence inside the nucleus after its ectopic expression; and failed to localize in mitochondria. On the other hand, ectopically expressed DNMT1-isoform3 targeted itself to mitochondria and subsequently methylated CpG regions in the mitochondrial genome. In addition, overexpression of DNMT1-isoform3 affected mitochondrial biology and regulated its function. Under different conditions of oxidative and nutritional stress, this isoform was down-regulated, resulting in hypomethylation of mitochondrial genome. Our study reveals how DNMT1-isoform3, instead of isoform1, is responsible for mtDNA methylation, influencing its biology.

## Introduction

Nuclear genome methylation in CpG regions is widely studied and is generally considered as a mark of transcriptional silencing. On methylation, MBD (Methyl CpG Binding Domain) proteins either recruit corepressor complex or directly inhibit transcription factor binding, ultimately leading to transcription repression^1^.

The presence and absence of this epigenetic modification in mitochondria has been under debate in literature for a long time^2–5^. The first study conducted 40 years ago reported an absence of mitochondrial DNA methylation in frogs and HeLa cells^3^. However, several studies thereafter suggested low levels of methylation in mitochondria in different species^6, 7^. In support of the occurrence of methylation, Shock *et al*.^8^. for the first time showed existence of a DNA methyltrasferase (DNMT1) inside mitochondria. After this, a number of reports in favor of this epigenetic modification continued to increase; and found differential methylation at certain CpG regions of mitochondrial genome in association with several pathophysiological conditions^9–12^.

However, none of these studies provided an insight into the mechanistic details of the epigenetic process and its role in mitochondrial biology. In the background of our previous studies^13, 14^ related to mitochondrial genomics and ROS mediated retrograde signaling and epigenetic modulation of nuclear gene expression^15^, we attempted to understand the process of mitochondrial epigenetic regulation. A direct approach of exogenous overexpression of specific isoforms of DNMT1 was used to find out if these localized in mitochondria and played a role in influencing its biology epigenetically.

## Results

### DNMT1-isoform3 not isoform1 localizes to the mitochondria

**Figure 1:**
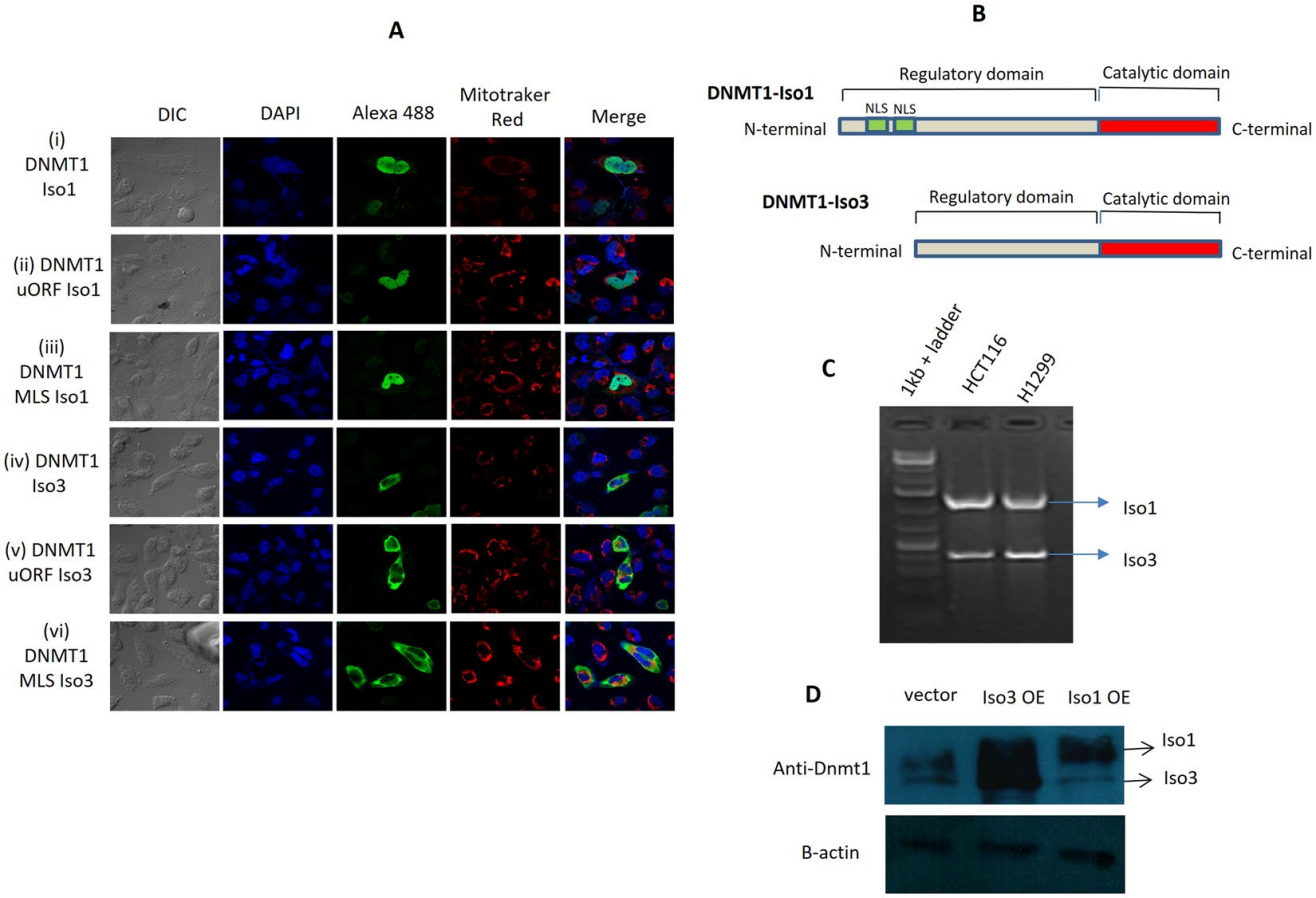
DNMT-isoform3 not isoform1 localizes to mitochondria. (**A**) Representative confocal images of H1299 cells showing subcellular localization pattern of exogenously over-expressed DNMT1-isoform1 and 3 with or without any additional localization signal sequence. Nucleus, overexpressed protein and mitochondria were stained with DAPI, Alexa488 and Mitotracker red respectively. (**B**) Structure of DNMT1-isoforms depicting presence of nuclear localization signal sequence elements exclusively in isoform 1. (**C**) 5′ RACE PCR product with isoform 1 & 3. (**D**) Western blot scans of DNMT1-isoform1 & 3 overexpression.

The isoform of DNMT1 that possessed an additional upstream open reading frame (uORF) which encoded for a mitochondrial targeting sequence (MTS) at the N-terminal, was suggested to facilitate localization of DNMT1 to mitochondria and methylate its genome^8^. However, the overexpression of this isoform under similar conditions (Fig. 1Aii) in our experiments failed to target DNMT1 in mitochondria. Experiments carried out to check the subcellular localization of DNMT1-isoform1 even with strong mitochondrial targeting sequence (Fig. 1Aiii), did not facilitate localization of DNMT1 within mitochondria, but showed an exclusive presence in the nucleus. On examination of DNMT1-isoform1 structure (Fig. 1B), we found multiple patches of nuclear localization signal sequences (NLS) that seemed to override the presence of MTS at N-terminal; and facilitated its targeting to the nucleus only.

Incidentally, we found another isoform of DNMT1 (Fig. 1Aiv), the DNMT1-isoform3 which localized to the mitochondria. The nuclear localization signal sequences were found to be absent in the isoform3, as it lacked the portion of N-terminal region and represented the shortest isoform of DNMT1. *In-silico* localization prediction by psortII program (Supplementary Table 1) found 8.7% of the isoform3 to localize in human mitochondria. Exogenously overexpressed DNMT1-isoform3 also showed a similar fraction localized in the mitochondria. In light of these findings, we performed 5′RACE experiments (Fig. 1C) and found that transcripts of both, DNMT1-isoform1 and 3 existed in two different cell lines (H1299, HCT116). Both of these isoforms were confirmed for their expression by Western blot (Fig. 1D).

### DNMT1-Isoform1 with uORF fails to localize in mitochondria at all analyzed time points

**Figure 2:**
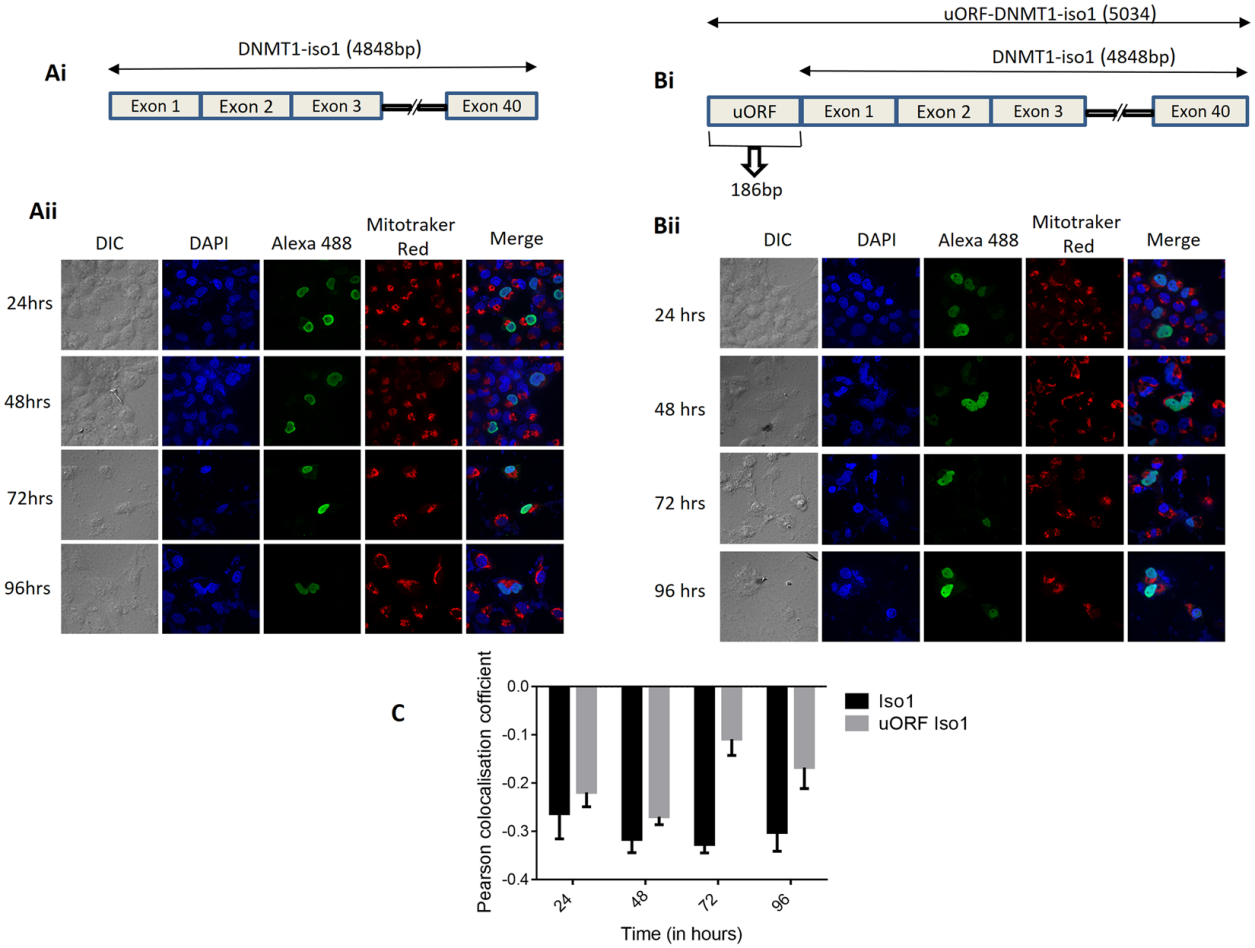
DNMT1-isoform1 fails to localize in mitochondria and exclusively localizes to the nucleus at all the analyzed time points. (**Ai-Bi**) Schematic diagram depicting the structure of: (**Ai**) DNMT1-iso1; (**Bi**) DNMT1-iso1 with uORF. (**Aii-Bii**) Representative confocal images of H1299 cells showing localization pattern of exogenously overexpressed: (**Aii**) DNMT1-isoform1 and (**Bii**) DNMT1-iso1 with uORF at four different time points (24, 48, 72, 96 hrs). (**C**) Pearson co-localization coefficient for mitochondrial localization of DNMT1-iso1 and DNMT1-iso1 with uORF.

Since it was DNMT1-isoform3 and not isoform1 that localized in the mitochondria, we further checked for the possibility of DNMT1 isoform1 to localize in mitochondria at different time points of its expression with and without additional localization signal sequence (uORF). DNMT1-isoform1 failed to localize in mitochondria at all the time points (24 hr, 48 hr, 72 hr, 96 hr) in growing cells in culture (Fig. 2A), even in presence of uORF, suggesting its exclusive localization in the nucleus (Fig. 2B). A negative co-relation for Pearson’s coefficient for mitochondrial localization of DNMT1-isoform1 with or without upstream localization signal sequences (Fig. 2C), confirmed the conclusions drawn.

### Subcellular localization of DNMT1-isoform3 varies with advancing time period of expression

**Figure 3:**
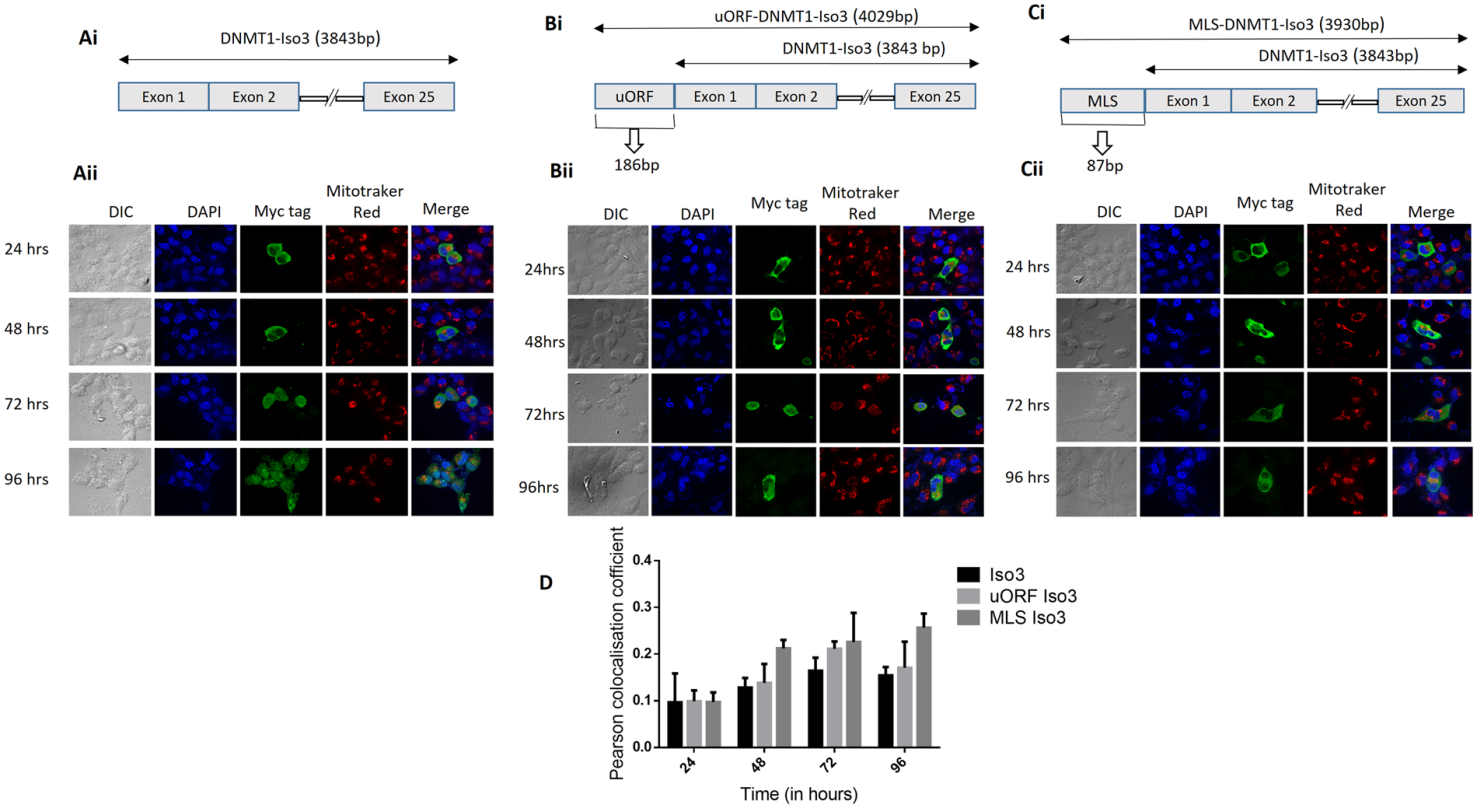
Subcellular localization of DNMT1-isoform3 varies with advancing time period of expression. (**Ai-Ci**) Schematic diagram depicting the structure of: (**Ai**) DNMT1-iso3; (**Bi**) DNMT1-iso3 with uORF; (**Ci**) DNMT1-iso3 with MLS. (**Aii-Cii**) Representative confocal images of H1299 cells showing localization pattern of exogenously overexpressed: (**Aii**) DNMT1-iso3; (**Bii**) DNMT1-iso3 with uORF; (**Cii**) DNMT1-iso3 with MLS at four different time points (24, 48, 72, 96 hrs). (**C**) Pearson co-localization coefficient for mitochondrial localization of DNMT1-iso3, DNMT1-iso3 with uORF and DNMT1-iso3 with MLS.

A change in the pattern of localization was observed for DNMT1-isoform3 between mitochondria and nucleus with advancing time after its exogenous expression (Fig. 3A). This isoform exclusively localized to mitochondria and cytosol at 24 and 48 hrs of expression. In the later hours (72 and 96 hrs), however, it localized in the nucleus and was gradually distributed throughout the cell. Inclusion of uORF sequence increased the potential of localization of the isoform3 in the mitochondria with concomitant decrease inside the nucleus (Fig. 3B). Further addition of a strong mitochondrial localization signal sequence to isoform3, prevented its localization in the nucleus even in later hours (72 and 96 hrs) of expression (Fig. 3C). The Pearson’s coefficient for mitochondrial localization showed a positive correlation for this isoform, which exhibited a further increase in presence of additional targeting sequence (Fig. 3D).

### Overexpression of DNMT1-isoform3 causes hypermethylation of mitochondrial DNA

**Figure 4:**
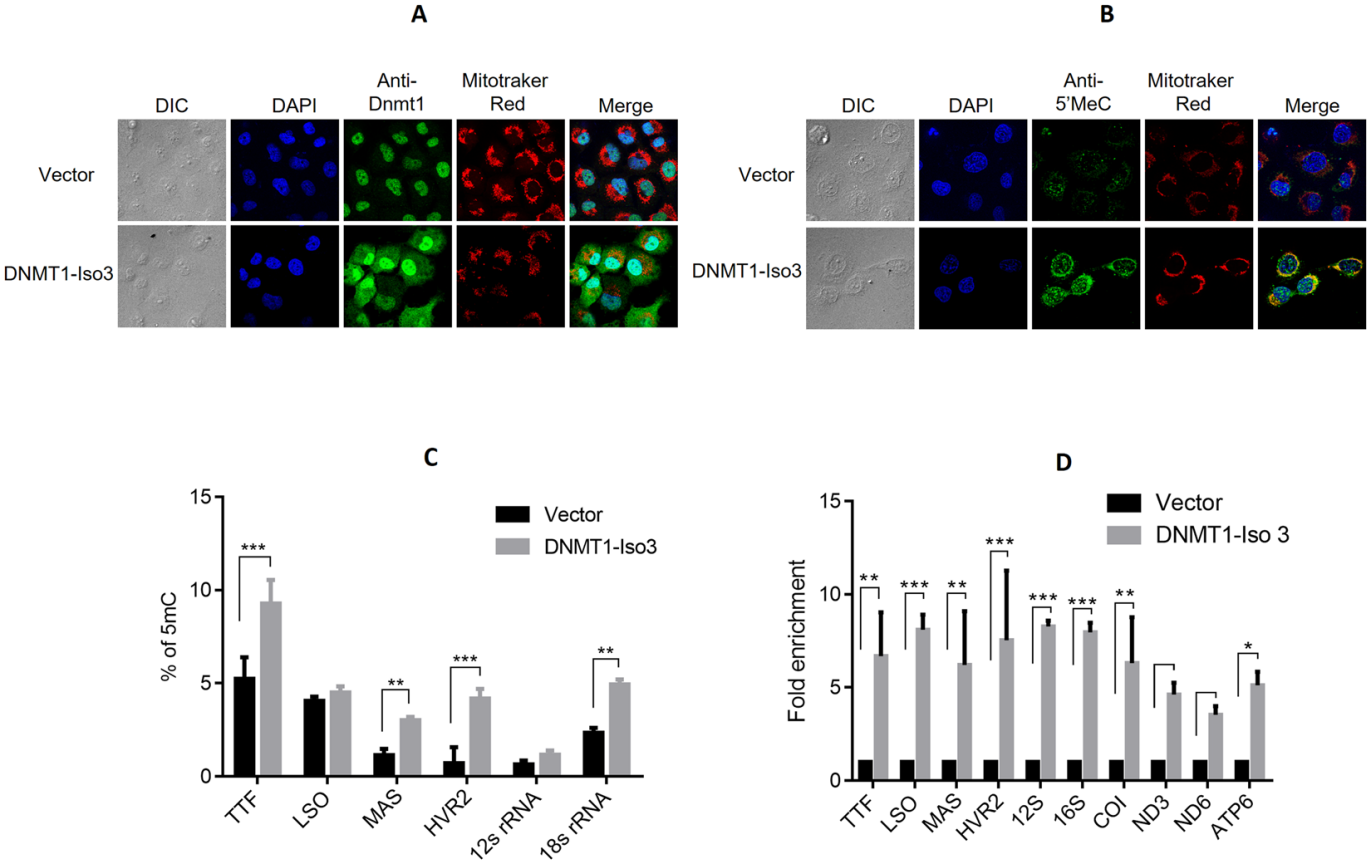
Overexpression of DNMT1-isoform3 causes hypermethylation of mitochondrial DNA. Representative confocal images of H1299 cells stably overexpressing DNMT1-iso3; (**A**) DNMT1 localisation pattern; (**B**) 5 mC staining; (**C**) % of 5 mC at defined CpG sites of mitochondrial genome resulting from overexpression of DNMT1-iso3; (**D**) Relative fold enrichment of selected mitochondrial regions for DNMT1-isoform3 binding by CHIP analysis.

Next, we expedited if the isoform3 present in mitochondria methylated its genome. For this, DNMT1-isoform3 was stably overexpressed in H1299 cells (Fig. 4A) and global mtDNA methylation analysis carried out by using 5 methyl cytosine (5 mC) antibody. In comparison to mock cells, the mitochondria of DNMT1-isoform3 overexpressing cells showed an increase in staining with 5 mC (Fig. 4B). The status of methylation of mtDNA was further confirmed by evaluating six different CpG positions located in the functionally important regions of mitochondrial genome, and finding an increase in methylation levels on overexpression of DNMT1-isoform3 (Fig. 4C). For final confirmation and validation, binding of DNMT1-isoform3 on mitochondrial DNA was checked by CHIP assay. All the regions screened (TTF, LSO, MAS, HVR2, 12s rRNA, 16s rRNA, CO1, ND3, ND6 and ATP6), which included the 6 hypermethylated sites as well, showed a significant increase in the enrichment of mtDNA fragments on overexpression of DNMT1-isoform3 (Fig. 4D). Overall, our data suggests that DNMT1-isoform3 binds to mtDNA and causes hypermethylation of the mitochondrial genome.

### Exogenous overexpression of DNMT1-isoform3 affects mitochondrial biology

**Figure 5:**
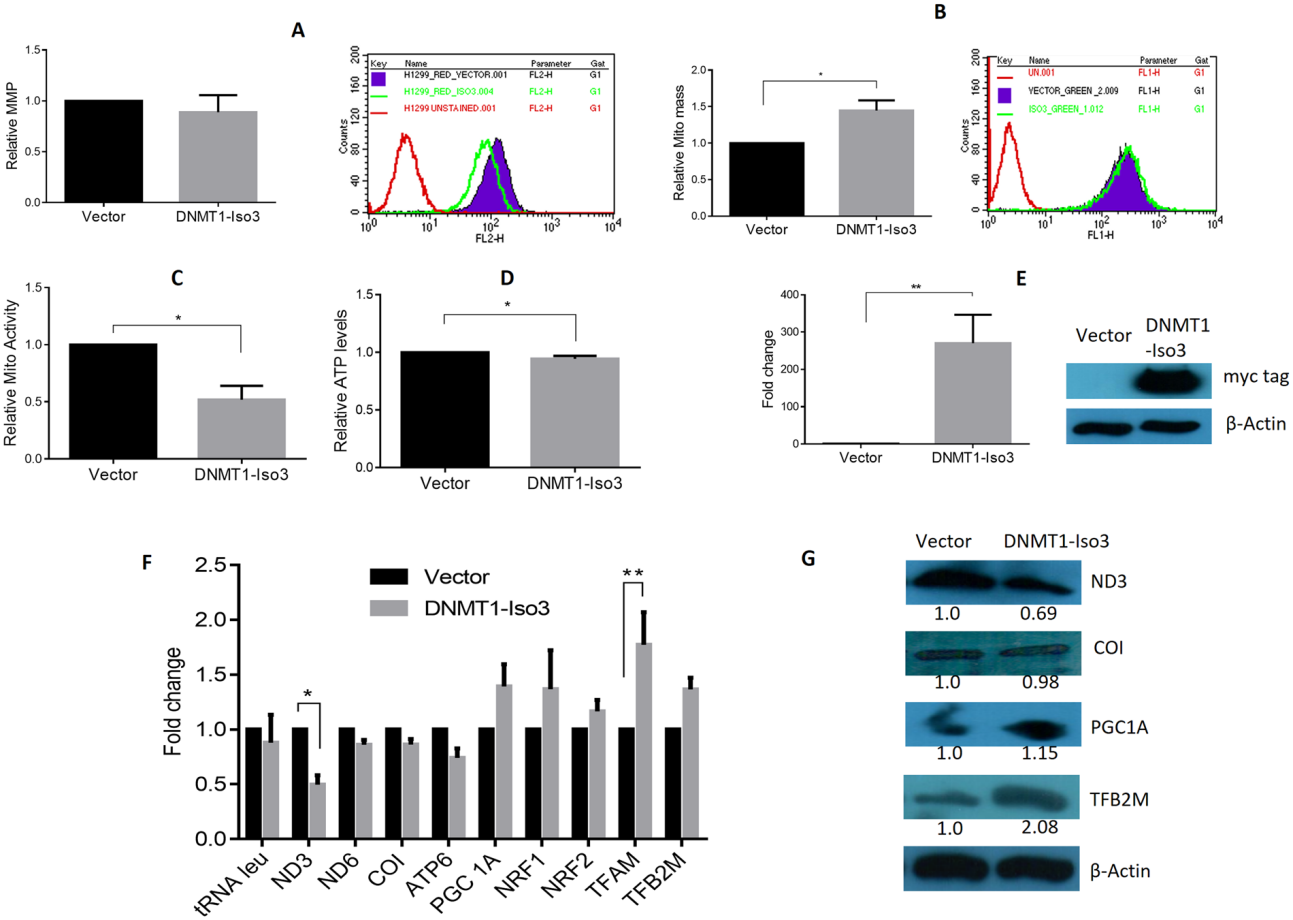
Exogenous overexpression of DNMT1-isoform3 affects mitochondrial biology. Representative screen shots and bar diagram for relative: (**A**) mitochondrial membrane potential; (**B**) mitochondrial mass; (**C**) mitochondrial activity; (**D**) ATP levels on exogenous overexpression of DNMT1-isoform3 in H1299 cells. (**E**) The real time and Western profile of DNMT1-iso3 overexpressing cells. (**F**) Semi-quantitative gene expression profile and (**G**) Western blot scans of selected mtDNA encoded and nuclear genome encoded mt genes in overexpressing DNMT1-iso3 H1299 cells. Relative fold of expression of the analyzed protein was calculated by densitometry analysis on normalization with respective β-actin expression and indicated (in values) just below the scans. The western blot scans were cropped for presentation in the main figure, the original scans are provided in the supplementary information.

As it became clear that it was only the isform3 that localized to mitochondria and methylated its genome; we sought to determine the role of this epigenetic process in the mitochondrial biology. After the overexpression of DNMT1-isoform3 (Fig. 5E), we assessed intermediate phenotypes of mitochondrial biology. There was a decrease in mitochondrial membrane potential (Fig. 5A) and an increase in mitochondrial mass (Fig. 5B), along with a significant decrease in mitochondrial activity (Fig. 5C) and ATP production (Fig. 5D) in comparison to mock transfections. We observed no significant change in the ROS levels under these conditions (Supp. Figure 2A). We then assessed the expression profile of mitochondrial genome encoded mitochondrial genes (ND3, ND6, CO1, ATP6, tRNAleu) and a set of nuclear genes involved in mitochondrial biogenesis (PGC1 A, NRF1, NRF2, TFAM, TFB2M), using real time qPCR analysis. The isoform3 overexpressed cells exhibited more or less, a decrease in expression of mitochondrial genome encoded oxidative phosphorylation genes (Fig. 4F), however, the nuclear genome encoded mitochondrial biogenesis genes were found to be upregulated. The protein expression assessed in Western blots of the representative genes (ND3, COI, PGC1A and TFB2M) confirmed the observations made in real-time expression profiles (Fig. 4G). These results together suggest that the overexpression of DNMT1-isoform3 affects mitochondrial biology by modulating the expression of nuclear and mitochondrial genome encoded genes.

### The mitochondrial genome is hypomethylated under conditions of nutritional and oxidative stress

**Figure 6:**
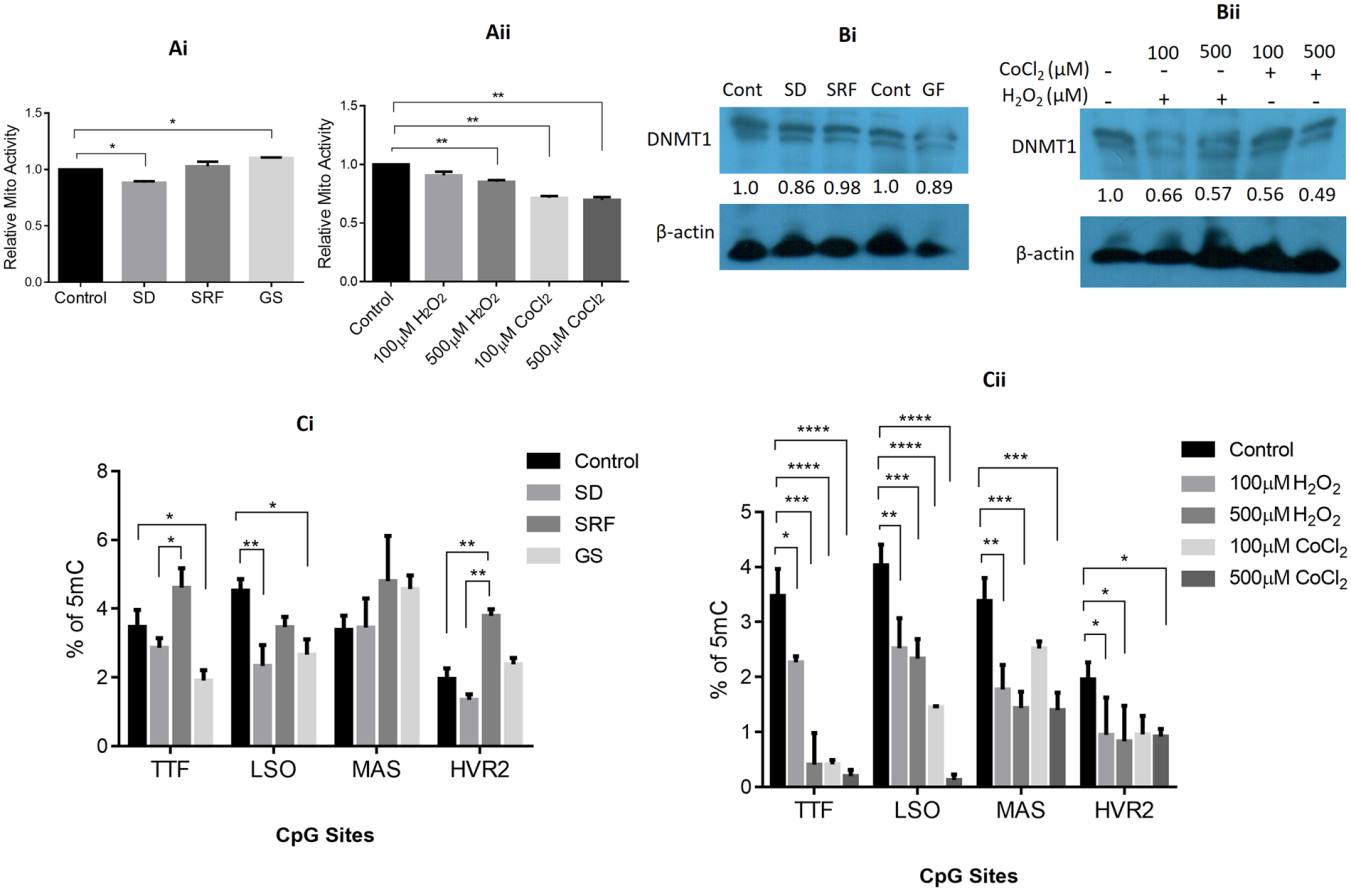
Mitochondrial DNA is hypomethylated under various conditions of nutritional and oxidative stress. (**A**) Relative mitochondrial activity in H1299 cells under different conditions of (i) nutritional and (ii) oxidative stress. (**B**) Western Blot scans of DNMT1 isoforms expression profile under (i) nutritional and oxidative (ii) stress conditions. (**C**) % of 5 mC at certain defined CpG sites of mitochondrial genome resulting from nutritional (i) and oxidative stress (ii) conditions respectively. SD, SRF and GF stands for serum deprivation, serum re-fed and glucose starvation respectively. H_2_O_2_ and CoCl_2_ treated and non-treated are indicated with (+) and (−) sign respectively. Relative fold of expression of DNMT1-isoform3 is calculated by densitometry analysis on normalization with respective β-actin expression and indicated (in values) just below the DNMT1 scans.

During various pathophysiological conditions of nutritional and oxidative stress, cells are subjected to several metabolic and epigenomic changes^16^. Here, we attempted to understand the effect of these conditions on the process of mtDNA methylation. We checked mitochondrial activity (Fig. 6Ai) of the cells in response to these conditions and observed that under glucose starvation (GS), the mitochondrial activity increased; while, under serum deprivation (SD), there was a decrease in mitochondrial activity, which was restored when cells were supplemented with serum (SRF). The ROS levels were elevated under both nutritional and oxidative stress conditions. Under nutritional stress, the glucose starvation condition showed a significant increase in ROS levels. Similarly, on serum starvation the increased level was restored to normal levels when cells were supplemented with serum (Supp. Figure 2B). Under oxidative stress conditions, the ROS levels were higher in H_2_O_2_ than CoCl_2_ treatment (Supp. Figure 2C). Western blot analysis revealed (Fig. 6Bi) downregulation in the expression profile of DNMT1-isoform3 accompanied with hypomethylation (Fig. 6Ci) at the selected CpG sites within TTF, LSO, MAS and HVR2, 12s and 16s rRNA regions in mtDNA. We then proceeded to identify the status of mtDNA methylation under oxidative stress conditions. There was a significant decrease in mitochondrial activity (Fig. 6Aii) and in the expression of DNMT1 isoforms (Fig. 6Bii). Along this, mitochondrial genome showed intensive hypomethylation (Fig. 6Cii) at all the analyzed CpG regions (TTF, LSO, MAS, and HVR2, 12s and 16s rRNA). These results together suggest that mtDNA is hypomethylated under conditions of nutritional and oxidative stress.

## Discussion

Our study for the first time provides a direct experimental proof of an isoform of DNMT1 methylating mitochondrial genome and influencing mitochondrial biology. We propose that it is DNMT-isoform3 instead of DNMT-isoform1 which localizes to mitochondria and initiates the process of methylation. An earlier report^8^ had suggested that DNMT-isoform1 with upstream 186 bp (uORF) sequence as a part of the transcript, had the potential to localize in mitochondria. The localization to the mitochondria was proposed to be possible due to the uORF shown to code for the region that acted as a mitochondrial localization signal sequence, enabling the reporter construct with GFP to translocate in mitochondria. Although, we found this upstream sequence as a part of the transcript, however, fusing this uORF to the main ORF of DNMT1 and then the exogenous expression of this N terminal extended DNMT1, failed to localize DNMT-isoform1 to mitochondria. Even DNMT1 cloned downstream to the strong mitochondrial localization signal sequence, failed again to localize in mitochondria and was exclusively translocated to the nucleus. Finding the N terminal region of DNMT1-isoform1 rich in arginine and lysine amino acid residues, known for their strong nuclear localization potential^17^, in all probability, prevented the localization of DNMT1-isoform1 to mitochondria. The obvious question as to how this basic epigenetic modification was happening in mitochondria, remained to be addressed.

The uniprot database when examined for the DNMT1 isoforms, showed 3 isoforms for DNMT1. Of these, isoform3 (Uniprot ID: P26358-3) was the shortest and lacked initial 15 exons coding for NLS of isoform1. The isoform3 when exogenously overexpressed, localized in mitochondria, subsequently methylating the cytosine residues in the mitochondrial genome. This was confirmed by evaluating the status of methylation at 6 out of 23 *in silico* screened sites with sequence “CCGG” (a recognition sequence for the pair of methyl sensitive restriction enzyme- MspI and HpaII) to assess their methylation status using methyl sensitive restriction qPCR (MSRP); and chosen on the basis of their location within important regulatory regions (TTF: transcription termination factor, LSO: L-stand origin of replication, MAS: membrane attachment site, HVR2: hypervariable region2, 12s rRNA, 16s rRNA) involved in transcription and replication of mtDNA with a possible role in mitochondrial function. Further confirmation was obtained through CHIP assay, where DNMT1-isoform3 was found bound to the mtDNA. The resultant effect of overexpression of DNMT1-isoform3 on the mitochondrial dysfunction was evident from the decrease in mitochondrial membrane potential, ATP production and the expression profile of mitochondrial genome encoded OXPHOS related genes. At the same time, an increase in the expression of nuclear genome encoded mitochondrial biogenesis genes with a concomitant increase in mitochondrial mass, probably indicated an adaptive response to maintain homeostasis^18^. The homeostatic response was evident from the observations made over a period of time (1month) from DNMT-isoform3 stably transduced cells, showing the restored status of mitochondrial mass and potential. We believe that the acute mitochondrial dysfunction that resulted in a drop in ΔΨ could prevent mitochondrial Ca^2+^ uptake and generate an elevated cytosolic Ca^2+^ levels. This in turn could activate calcineurin (a phosphatase). It is known that the activated calcineurin governs diverse cellular outcomes, resulting in transcription activation of a large set of genes involved in cellular metabolism, mitochondrial transcription and biogenesis^19^ Similarly, the drop in ATP levels is known to activate AMPK by phosphorylation that regulate the expression of mitochondrial biogenesis genes^20^.

Knowing that the mitochondria results in a rapid change in its structure and function^21^ under various pathophysiological conditions and stress, we attempted to understand how mtDNA methylation was affected in response to nutritional and oxidative stress. We evaluated the status of methylation at the studied CpG sites and observed that mtDNA was hypomethylated with concomitant downregulation of DNMT1-isoform3. Interestingly, it was observed that the hypomethylation under the conditions of oxidative stress was more extensive in comparison to nutritional stress at these analyzed CpG positions. To our surprise, these sites behaved differently in terms of their methylation levels in a given particular condition; for example, the hypomethylation at TTF site in mitochondrial genome under oxidative stress conditions was most prominent among other analyzed sites. The TTF site located immediately downstream of the 3′ end of the 16S rRNA within the tRNA^Leu(UUR)^ gene; acted as a binding site for mTERF, that was shown to arrest *in vitro* mitochondrial RNA polymerase (mtRNAP) progression from Heavy Strand Promoter I^22^. Similarly, CpG site within LSO region, a part of the template sequence 3′-GGCCG-5′ located immediately adjacent to the stem loop structure and known to be essential for efficient replication^23^, was also hypomethylated, though not to the same extent as was observed for TTF site. Whereas, CpG position in MAS, the region of mitochondrial genome associated with the inner mitochondrial membrane^24^, showed hypermethylation on glucose starvation; without showing a change under serum deprivation condition, unlike other studied sites which were hypomethylated.

Of note, the evaluated CpG sites had physiological importance as these were located within regulatory regions involved in transcription and replication of mtDNA. Given that the frequency of methylation of mitochondrial genome was different at all these analyzed CpG positions, it is pertinent to identify more of such regions which are differentially methylated under several disease and physiological conditions. The regulation of the mtDNA methylation, apart from DNMT1 expression, has been suggested to be influenced by the availability of S-adenosyl methionine (SAM). The lower levels of SAM in cytosol was correlated with the lower levels of mtDNA methylation in Down Syndrome (DS) patients^25^; while, the higher levels of SAM generated due to overexpression of SLC25A26 gene (encoding for the mitochondrial carrier that catalyzes the import of SAM into the mitochondrial matrix), promoted hypermethylation of mtDNA, leading to decreased expression of key respiratory complex subunits, reduction of mitochondrial ATP and release of cytochrome c^26^. These observations highlight the importance of SAM availability, suggesting that its synthesis and transport to mitochondria could represent key regulatory steps of mtDNA methylation. Differential methylation of mitochondrial genome have been studied in association with several disease and health conditions^9, 12, 27–29^; and more of such studies are required to understand the biological relevance of the methylome landscape of the mitochondrial genome under diverse pathophysiological conditions, for a better prognosis of the mitochondria associated diseases.

**Figure 7:**
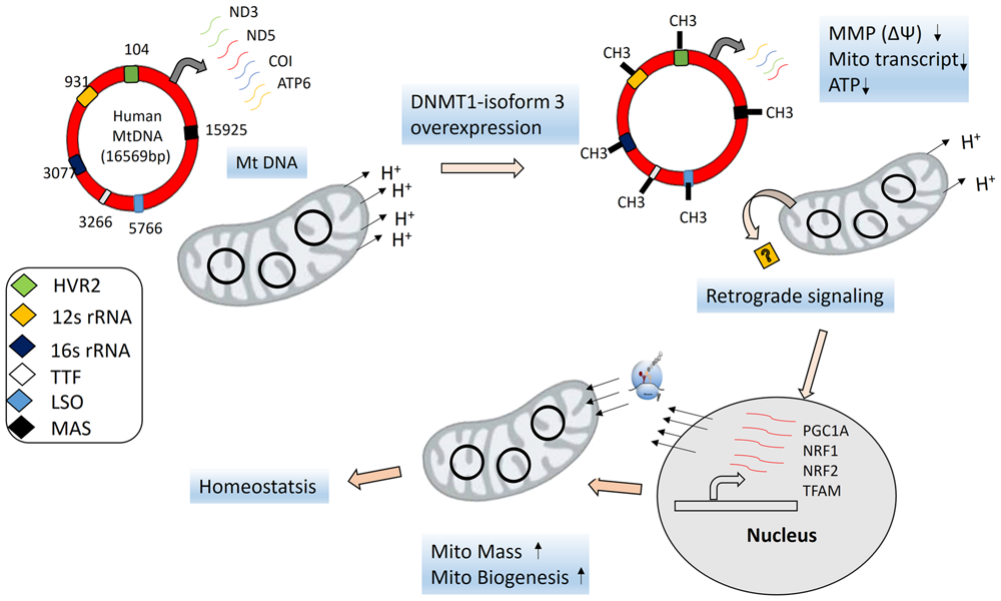
The proposed model depicting DNMT1-isoform3 function in mitochondrial biology. DNMT1 isoform3 methylates mitochondrial DNA and causes mitochondrial dysfunction, evident from a decrease in MMP, mitochondrial transcripts (ND3 ND6, COI, ATP6) and ATP levels. The acute mitochondrial dysfunction is communicated to nucleus through retrograde signaling, which results in upregulation of mitochondrial biogenesis genes (PGC1A, NRF1, NRF2, TFAM, TFB2M) and an increase in mitochondrial mass to maintain the homeostatsis. The six diagonals with different color codes represents the CpG sites (TTF: transcription termination factor at 3266, LSO: L-stand origin of replication at 5766, MAS: membrane attachment site at 15925, HVR2: hypervariable region2, 12s rRNA at 104, 16s rRNA at 3077 positions in mtDNA) located within the important regulatory regions involved in mitochondrial transcription and replication.

To sum up, we have demonstrated that it is the DNMT1-isoform3 not isoform1 which localizes in mitochondria and subsequently methylates mtDNA. The epigenetic process of mtDNA methylation modulates mitochondrial biology (Fig. 7) and has biological relevance as it is differentially hypomethylated under different conditions of oxidative and nutritional stress.

## Materials and Methods

### Plasmid construction and SDM

DNMT1 isoforms 1 and 3 were amplified using pcDNA3/myc-DNMT1 vector (obtained from addgene). DNMT1 isoforms were cloned in pcDNA3.1myc/his A (-) vector (Invitrogen #V855-20) at EcoR1 and Kpn1 restriction sites. DNMT1-isoforms in frame with uORF (upstream open reading frame) were generated by using a three-step cloning strategy: (i) where the sequence encoding uORF was amplified using H1299 genomic DNA and was cloned at Nhe1 and EcoR1 sites to generate pcDNA3.1-uORF construct, (ii) DNMT1 isoforms were cloned in the recombinant construct (pcDNA3.1-uORF) at EcoR1 and Kpn1 restriction sites, (iii) followed by deletion of EcoR1 restriction site located in-between uORF and DNMT1 isoforms, by site directed mutagenesis [using Q5® site-directed mutagenesis kit (NEB #E0554S)] to obtain the resultant constructs of pcDNA3.1 with uORF in frame with DNMT1 isoforms. For mitochondrial localization, DNMT1 isoforms were cloned into Sal1 and Not1 restriction sites of pCMV-myc-mito (Invitrogen #V822-20) vector with N-terminal mitochondrial targeting sequence (MTS) and C-terminal myc epitope. For stable cell line generation, DNMT1-isoform3 was cloned at EcoR1 and Xba1 restriction sites in plvx-puro lentiviral vector (Clontech #632164). The generated constructs were confirmed by sequencing, using ABI 3130xl genetic analyzer.

### In-silico localization prediction

The cDNA sequence of DNMT1-isoforms 1 or 3 fused in frame with, either uORF of DNMT1 gene or mitochondrial targeting sequence of human cytochrome c oxidase subunit VIII at their 5′end, were assessed for their potential to localize in different subcellular compartments, using PSORT II localization prediction program (http://psort.hgc.jp/form2.html).

### Cell culture and stress exposure

H1299 and HCT116 cell lines were grown in DMEM medium supplemented with 10% Fetal Bovine Serum (FBS), containing appropriate antibiotics (Pencillin and Streptomycin) in a humidified incubator at 37 °C and 5% CO_2_. Nutritional stress was induced by starving the cells in culture either to low serum (0.5% FBS) or low glucose (1 mM) for 18 h; whereas, oxidative stress was induced by treating the cells in culture with different doses of H2O2 (100 μM and 500 μM) and CoCl2 (100 μM and 500 μM) for 18 h. Cells were transfected with the desired constructs at 70–80% confluency using Lipofectamine 3000 reagent (TS #L3000015).

### Virus production and stable cell generation

Stable cells were generated using lentiviral particles. Lentiviral particles were produced by co-transfecting HEK293-T cells with transfer plasmid along with psPAX2 and pMD2.G, using Lipofectamine 3000 reagent. Fifteen hours after transfection, the medium was replaced with fresh DMEM. Supernatant containing virus particles was collected twice at 36 h and 50 h after transfection. The viral supernatant was filtered through a 0.45-µm filter and kept either at 4 °C to use on the same day or stored at −80 °C for long term storage. For virus infection, H1299 cells were plated in polybrene (8 µg/ml) containing DMEM and simultaneously infected with virus supernatant for 12 h and kept in an incubator at 37 °C. Cells were selected after 24 h of infection with puromycin (2 µg/ml) containing medium. The ectopic expression of the puromycin resistant cells was monitored by RT PCR analysis or Western blot.

### RNA Isolation and Real-Time PCR

Total cellular RNA was isolated using Tri Reagent (Sigma #93289-25 ML). Reverse transcription reaction was performed for cDNA conversion, using Revert-Aid reverse transcription kit (Thermo scientific #K1621). PCR reactions were carried out with 75 ng of cDNA as template, using PowerUp SYBR Green Master Mix (Thermo Scientific #A25742) on Real Time Detection System (CFX-96, Biorad #1855195). All primers used in this study are listed in the Supplementary information (Suppl. Table 2).

### 5′ Rapid amplification of cDNA ends (5′ RACE)

5′ RACE experiment was carried out to identify 5′end of DNMT1 gene using SMARTer RACE 5′/3′ Kit (Clontech #634858). Briefly, RNA was converted to cDNA using 5′-CDS A as forward primer and gene specific internal primer as reverse primer (sequence detail provided in Supplementary Table 2). This cDNA was used to amplify 5′region of DNMT1. Amplicons were cloned into pJET1.2 vector (Fermentas #K1231) and confirmed by sequencing (primer sequence provided in Sup. Table 2).

### Confocal Microscopy and Immunostaining

Transfection with different constructs was performed on the cells which were seeded on cover slips. After 48 h of transfection, cells were incubated with 1 nM Mitotracker Red in DMEM medium for 20 min at 37 °C, to stain mitochondria. For DNMT1 immunostaining, cell fixation was done with 3.7% paraformaldehyde solution in PBS for 20 min. Blocking and permeabilization was done together in PBS containing 5% BSA and 0.1% triton X 100. Permeabilized cells were incubated overnight with desired antibodies at 4 °C, followed by washing and incubation with Alexa-488 linked secondary antibody at room temperature for 1 h. Thereafter, nuclei were stained with 1 nM DAPI in PBS for 20 min at room temperature. Mounting was done in presence of prolong gold anti-fade reagent (Invitrogen, USA). Images were captured on Andor Spinning Disc confocal microscope equipped with iXon Ultra 897 EMCCD camera with 60x oil immersion objective lens. Pearson coefficient for mitochondrial co-localization was calculated for multiple images, using NIS viewer software. For 5 methyl cytosine immunostaining, cell fixation was carried out with ice-chilled methanol at −20 °C for 10 minutes, followed by permeabilization with ice-chilled acetone for 1 min at −20 °C. Further, cells were rehydrated in PBS and treated with 2 N HCl for 30 min at 37 °C. Cells were then washed twice with 0.1 M borate buffer (pH 8.5) and blocked with 1% BSA in PBS. After this step, cells were stained with 5 methyl cytosine antibody (Sigma #SAB4800001**)** at room temperature in dark for 1 h, followed by washing and incubation with Alexa-488 linked secondary antibody at room temperature for 1 h. Later steps were similar as mentioned above for DNMT1 immunostaining.

### Methylation analysis

Genomic DNA was isolated using QIAamp DNA Blood Mini Kit (50) from Qiagen. Mitochondrial whole genome sequence was screened for the sequence “CCGG” (a recognition sequence for the pair of methyl sensitive restriction enzyme- MspI and HpaII). These CpG sites when analyzed *in silico* turned out to be 23 in number in whole mitochondrial genome. Of these, 6 sites (TTF: transcription termination factor at 3266, LSO: L-stand origin of replication at 5766, MAS: membrane attachment site at 15925, HVR2: hypervariable region2, 12s rRNA at 104, 16s rRNA at 3077 positions) located within the mitochondrial genome in important regulatory regions involved in transcription and replication of mtDNA with a possible role in mitochondrial function, were chosen to assess their methylation status using EpiJET DNA Methylation Analysis Kit (MspI/HpaII, #1441). Briefly, 500 ng of total genomic DNA was digested with MspI and HpaII restriction enzymes at 37 °C for 4 h, followed by restriction enzyme inactivation by heating at 80 °C for 20 minutes. 25 ng of each MspI and HpaII digested DNA was used as a template to carry out Real-time PCR for the above mentioned CpG positions, using mitochondrial DNA specific flanking primers (the sequence of all the primers used are mentioned in Supp. Table 2).

### CHIP Assay

Chromatin immunoprecipitation was carried out by ChromaFlash High Sensitivity CHIP Kit from Epigentek (#P-2027). Briefly, cells in culture were harvested and washed with PBS twice. For cross-linking, cells were then incubated for 10 min with 1% formaldehyde solution containing 10 ml DMEM medium, on a rocker. 1 ml of 1.25 M glycine was added and centrifuged at 1000 RPM for 5 min. Cell pellet was washed with ice-chilled PBS and lysed on ice for 10 min. Chromatin collected after centrifugation; incubated with CHIP buffer for 10 min was sheared using probe-based sonication (Branson sonifier) for 10 cycles of 15 sec ON and 40 sec OFF at 25% power output. Chromatin was then processed according to manufacturer’s recommendation. De-crosslinked DNA was used as a template to carry out real time PCR to assess mtDNA binding sites of DNMT1 at multiple positions of the mitochondrial genome.

### ATP Production

The cellular ATP levels were measured using ApoSENSOR™ ATP Cell Viability Bioluminescence Assay Kit (Biovision #K254), according to manufacturer’s instructions. In brief, 10000 cells were lysed in buffer supplied with the product and assayed for ATP levels. The assay utilizes the enzyme luciferase to catalyze the formation of light from ATP and luciferin, where the light was measured using a luminometer.

### Western Blotting

For immunodetection, samples prepared by lysis of cell pellets in RIPA buffer and estimated for the concentration of proteins by bicinchoninic acid assay using BSA as a standard (Pierce #23227), were separated on 8–12% SDS–PAGE as required. The gel was then transferred on to a nitrocellulose membrane (NCM) via wet transfer method and was further processed using standard methods. The antigens were detected using primary antibodies (Anti-DNMT1 #ab13537, Abcam; beta-actin #A2228-100UL, Sigma; Anti-ND3 #ab170681 abcam; PGC1A #SC13067 Santa Cruz; TFB2M #H00064216-M01, Noche biologicals), followed by secondary immunoglobulins conjugated to HRP (1:10,000, Sigma). ECL reagents were used for visualization (Luminata Forte Western HRP substrate, Millipore).

### Flow Cytometry

Cells were harvested; washed with PBS twice and stained with Mitotracker Red CMX ROS (Invitrogen #M7512) to assess mitochondrial membrane potential or Mitotracker Green (Invitrogen #M 7514) to assess mitochondrial mass at a working concentration of 100 nM in complete DMEM medium for 30 min at 37 °C. For ROS assessment, cells were re-suspended in 1 ml of fresh complete medium, containing a final concentration of 5 μM of DCFH-DA (Sigma #D6883) and incubated for 45 minutes at 37 °C. Further, the stained cells with respective dyes were centrifuged; washed with PBS twice and suspended in PBS. These cells were run on flow cytometer and fluorescence recorded at respective channels. Data was analyzed by using “Cellquest Pro” software provided with the system from BD biosciences, USA.

### Statistics

Statistical analysis was performed using Graph Pad Prism software. Data was represented as mean ± SD. Multiple comparison analysis was done by using 2 way ANOVA Sidak’s test while for comparison of two samples unpaired t-test was used. A difference was considered significant when *P < 0.05 or **P < 0.01 or ***P < 0.001.

## Electronic supplementary material

Supplementary information
