# Supplementary material for: DNA Methyltransferase1 (DNMT1) Isoform3 methylates mitochondrial genome and modulates its biology
Source: Sci Rep. 2017 May 8;7:1525. doi: 10.1038/s41598-017-01743-y (PMC5431478; doi:10.1038/s41598-017-01743-y)
Supplement: Supplementary file 1 — Supplementary information [file 41598_2017_1743_MOESM1_ESM.pdf]

DNA Methyltransferase1 (DNMT1) Isoform3 methylates mitochondrial genome and modulates its biology

Sunil Kumar Saini, Kailash Chandra Mangalhara, Gopinath Prakasam, RNK Bamezai*

National Centre of Applied Human Genetics, School of Life Sciences, Jawaharlal Nehru University, New Delhi-110067, India.

*Address correspondence: RNK Bamezai; E-mail: bamezai@hotmail.com

Supplementary Table 1: Subcellular localization prediction of DNMT1-isoforms.

S.No.	**DNMT1-isoforms with/without additional localization signal sequence**	**Localization prediction**	
1.	DNMT1-isoform1	82.6 %: nuclear<br>08.7 %: plasma membrane<br>04.3 %: cytoplasmic<br>04.3 %: cytoskeletal	
2.	DNMT1-isoform1 with uORF	78.3 %: nuclear<br>13.0 %: plasma membrane<br>08.7 %: cytoskeletal	
3.	DNMT1-isoform1 with MLS	65.2 %: nuclear<br>03.0 %: cytoskeletal<br>08.7 %: cytoplasmic<br>08.7 %: mitochondrial<br>04.3 %: plasma membrane	
4.	DNMT1-isoform3	78.3 %: nuclear<br>08.7 %: cytoplasmic<br>08.7 %: mitochondrial<br>04.3 %: plasma membrane	
5.	DNMT1-isoform3 with uORF	69.6 %: nuclear<br>13.0 %: cytoplasmic<br>08.7 %: mitochondrial<br>04.3 %: cytoskeletal<br>04.3 %: plasma membrane	
6.	DNMT1-isoform3 with MLS	56.5 %: nuclear<br>39.1 %: mitochondrial<br>04.3 %: cytoplasmic	

Supplementary Table 2: Information of the primers used in the study.

S. No.	Purpose of Primer	Primer Sequence	Reference
1	Primers for DNMT1 cloning		
	uORF NheI F	TTTGCTAGCATGGCCGGCTCCGTT	In this study
	uORF EcoRI R	TTTGAATTCCTCGGAGGCTTCAGCAGACG	In this study
	DNMT1-iso1 EcoRI F	TTTGAATTCATGCCGGCGCGTACCGCCCCA	In this study
	DNMT1-iso1 KpnI R	GGGCGGTACCGTCCTTAGCAGCTTCCTCCTCCTTTA	In this study
	DNMT1-iso3 EcoRI F	TTTGAATTCATGGCTCGCGCCAAAACAG	In this study
	DNMT1-iso3 KpnI R	GGGCGGTACCGTCCTTAGCAGCTTCCTCCTCCTTTA	In this study
	DNMT1-iso1 SalI F	TTAGTCGACATGCCGGCGCGTACC	In this study
	DNMT1-iso1 NotI R	TTTGCGGCCGCGTCCTTAGCAGCTTC	In this study
	DNMT1-iso3 SalI F	TTAGTCGACATGGCTCGCGCCAAAACAGTC	In this study
	DNMT1-iso3 NotI R	TTTGCGGCCGCGTCCTTAGCAGCTTC	In this study
	DNMT1 XbaI R	TTAATCTAGAGAGGAAGCTGCTAAGGAC	In this study
			In this study
2	Primers for SDM		
	DNMT1-iso1 EcoRI Deletion F	ATGCCGGCGCGTACCGCC	In this study
	DNMT1-iso3 EcoRI Deletion F	ATGGCTCGCGCCAAAACAGT	In this study
	DNMT1 EcoRI Deletion R	CTCGGAGGCTTCAGCAGACGCG	In this study
3	Primers for 5' RACE		
	5' CDS A F	Sequence not disclosed by manufacturer	
	UPM Short	CTAATACGACTCACTATAGGGC	
	DNMT1 internal R	CACACTGAAGCAGGTCAGTTTGTGCTGG	In this study
4	Primers for Real Time Quantification		
	DNMT1 F	GGCTGAGATGAGGCAAAAAG	In this study
	DNMT1 R	ACCAACTCGGTACAGGATGC	In this study
	HVR2 F	GCTCTCCATGCATTTGGTAT	In this study
	HVR2 R	AGGATGAGGCAGGAATCAAAG	In this study
	Transcription termination factor F	CACCCAAGAACAGGGTTTGT	In this study
	Transcription termination factor R	TGGCCATGGGTATGTTGTTAAG	In this study
	L- Strand origin F	CCCTAATCAACTGGCTTCAATCT	In this study
	L- Strand origin R	TACCAGCTCCGAGGTGATTT	In this study
	Membrane attachment site F	ATGGGCCTGTCCTTGTAGTA	In this study
	Membrane attachment site R	GGGTGCTAATGGTGGAGTTAAA	In this study
	COI F	TTCTGACTCTTACCTCCCTCTC	In this study
	COI R	TGGGAGTAGTTCCCTGCTAA	In this study
	ND3 F	CCACAACTCAACGGCTACATA	In this study

ND3 R	AGGAGGGCAATTTCTAGATCAAA	In this study
ND6 F	AGGATTGGTGCTGTGGGTGAAAGA	In this study
ND6 R	ATAGGATCCTCCCGAATCAACCCT	In this study
ATP6 F	TAGCCCACTTCTTACCACAAGGCA	In this study
ATP6 R	TGAGTAGGTGGCCTGCAGTAATGT	In this study
12s rRNA F	GGTCACACGATTAACCCAAGT	In this study
12s rRNA R	TGTTAAAGCCACTTTCGTAGTCTAT	In this study
16s rRNA F	GCCGCTATTAAAGGTTCGTTTG	In this study
16s rRNA R	CCTTTCGTACAGGGAGGAATTT	In this study
PGC1 A F	TGTCACCACCCAAATCCTTATTT	1
PGC1 A R	TGTGTCGAGAAAAGGACCTTGA	
NRF1 F	CCATCTG GTGGCCTGAAG	2
NRF1 R	GTGCCTGGGTCCATGAAA	
NRF2 F	ACACGGTCCACAGCTCATC	3
NRF2 R	TGTCAATCAAATCCATGTCCTG	
TFAM F	GAACAACTACCCATATTTAAAGCTCA	1
TFAM R	GAATCAGGAAGTTCCCTCCA	

Supplemetary Figure: 1

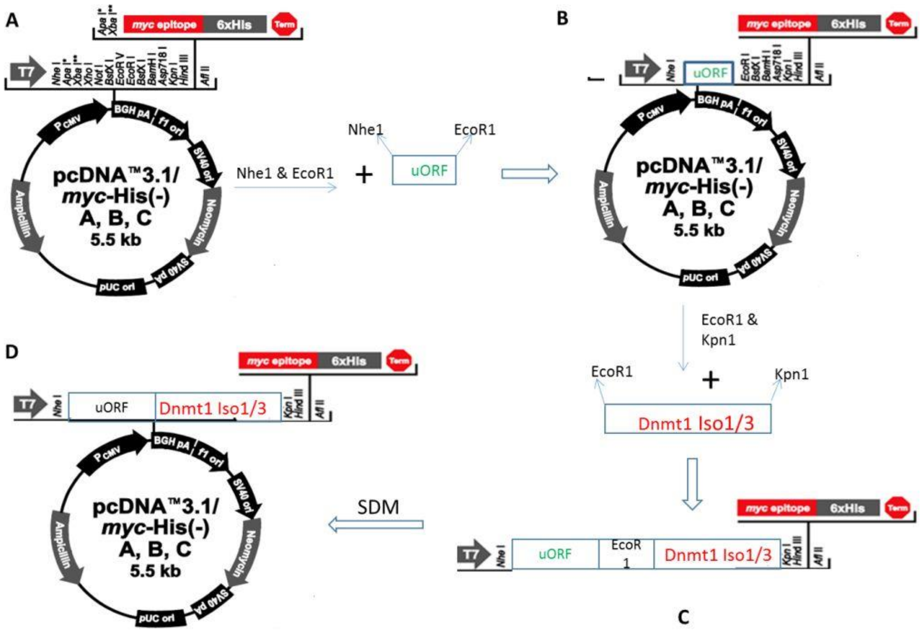

Figure S1: Cloning Strategy to clone DNMT1-Isoforms 1 and 3 with uORF sequence. (A) The vector backbone of pcDNA3.1-myc-his was double digested with NheI & EcoRI; similarly the PCR product for uORF sequence was digested with the same set of restriction enzymes. (B) Ligation of the digested uORF PCR product to the vector to generate pcDNA3.1-uORF-myc-his. (C) Digestion of (B) construct with EcoRI

and KpnI restriction enzymes and ligation with double digested (EcoRI and KpnI) PCR product of DNMT1-Isoforms 1 or 3 to generate pcDNA3.1 uORF-DNMT1-Isoforms 1 & 3 vectors. (D) Deletion by site directed mutagenesis of the EcoRI restriction site in-between uORF and DNMT1 sequence to generate the final clone of uORF in frame with DNMT1-Isoforms 1 and 3 independently.

Supplementary Figure: 2

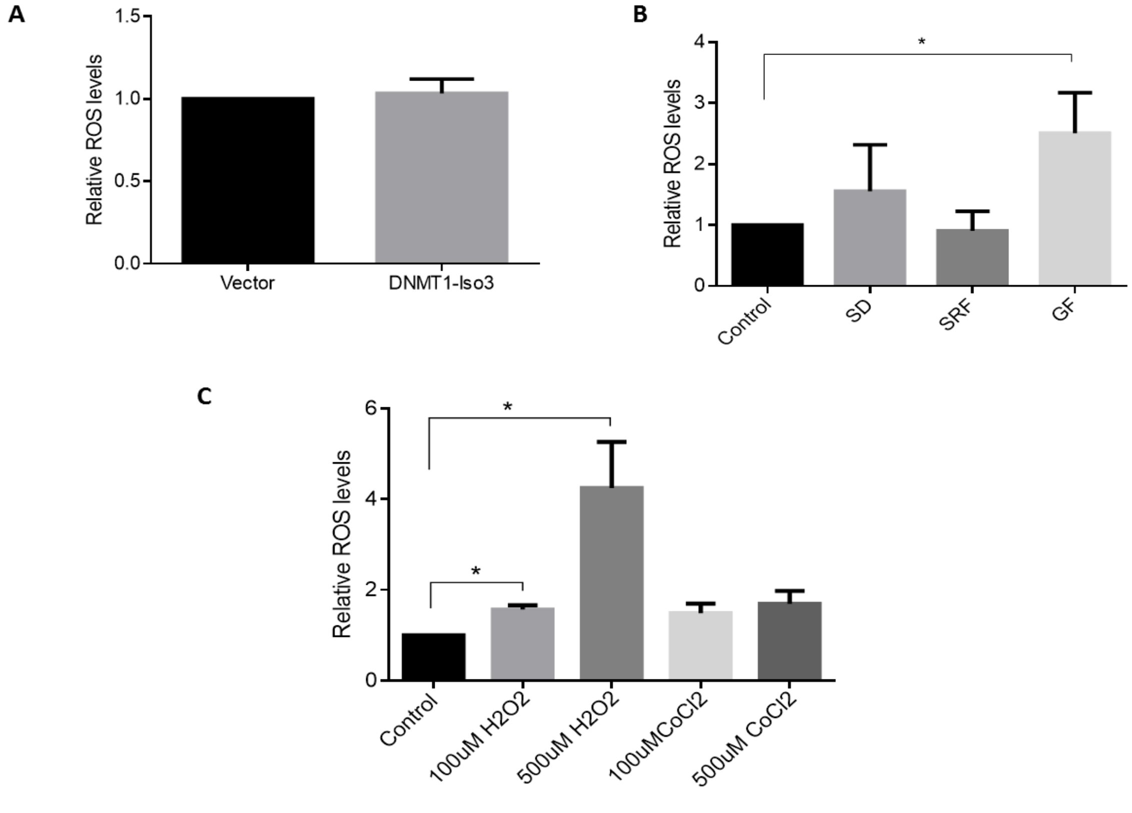

Figure S2: The cellular ROS levels in different experimental conditions. The ROS levels persisting in: (A) DNMT1-isoform3 overexpression; (B) Nutritional stress conditions; (C) Oxidative stress conditions. The SD, SRF and GF stands for serum deprivation, serum re-fed and glucose starvation respectively.

Raw Data for Figure 5E and 5G

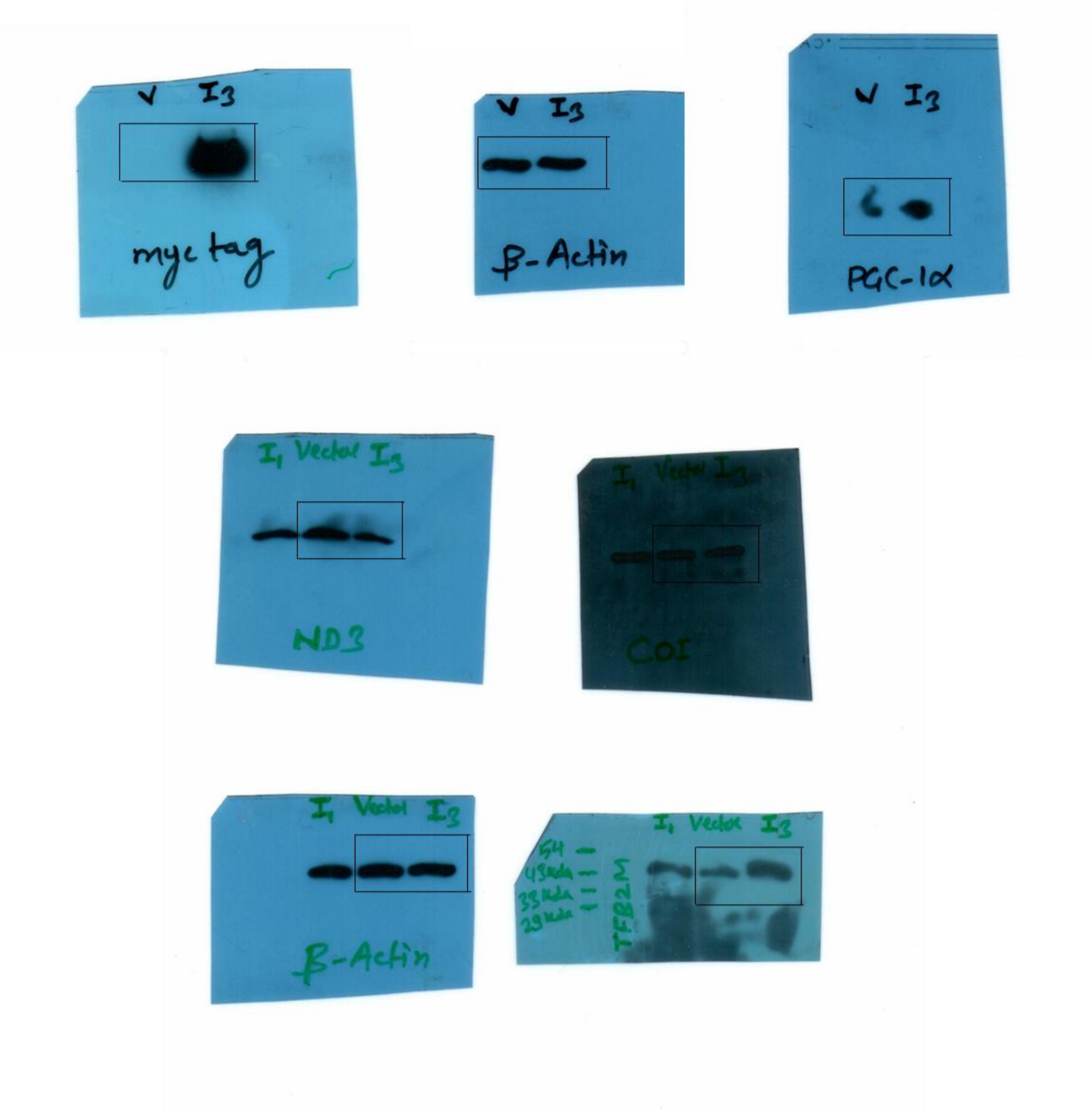

Supplementary References:

1 Liu, L. *et al.* LRP130 protein remodels mitochondria and stimulates fatty acid oxidation. *The Journal of biological chemistry* **286**, 41253-41264, doi:10.1074/jbc.M111.276121 (2011).

2 Chen, Y. *et al.* Synergistic Effects of Cilostazol and Probucol on ER Stress-Induced Hepatic Steatosis via Heme Oxygenase-1-Dependent Activation of Mitochondrial Biogenesis. *Oxidative medicine and cellular longevity* **2016**, 3949813, doi:10.1155/2016/3949813 (2016).

3 Wang, X. J. *et al.* Nrf2 enhances resistance of cancer cells to chemotherapeutic drugs, the dark side of Nrf2. *Carcinogenesis* **29**, 1235-1243, doi:10.1093/carcin/bgn095 (2008).
